# The impact of FGFR1 and FRS2α expression on sorafenib treatment in metastatic renal cell carcinoma

**DOI:** 10.1186/s12885-015-1302-1

**Published:** 2015-04-18

**Authors:** Thai H Ho, Xian-De Liu, Yanqing Huang, Carla L Warneke, Marcella M Johnson, Anh Hoang, Pheroze Tamboli, Fen Wang, Eric Jonasch

**Affiliations:** 1 Division of Hematology and Medical Oncology, Mayo Clinic, Scottsdale, USA; 2 Department of Genitourinary Medical Oncology, The University of Texas MD Anderson Cancer Center, Houston, USA; 3 Center for Cancer and Stem Cell Biology, Texas A&M Institute of Biosciences and Technology, Houston, USA; 4 Department of Biostatistics, The University of Texas MD Anderson Cancer Center, Houston, USA; 5 Department of Pathology, The University of Texas MD Anderson Cancer Center, Houston, USA

**Keywords:** FGF, Renal cell carcinoma, VEGF, Sorafenib

## Abstract

**Background:**

Angiogenesis plays a role in tumor growth and is partly mediated by factors in both the fibroblast growth factor (FGF) and vascular endothelial growth factor (VEGF) pathways. Durable clinical responses with VEGF tyrosine kinase inhibitors (TKIs) may be limited by intrinsic tumor resistance. We hypothesized that FGF signaling may impact clinical responses to sorafenib.

**Methods:**

Nephrectomy material was available from 40 patients with metastatic renal cell carcinoma (RCC) enrolled in a phase II clinical trial of sorafenib ± interferon (ClinicalTrials.gov Identifier NCT00126594). Fibroblast growth factor receptor 1 (FGFR1) and fibroblast growth factor receptor substrate 2 alpha (FRS2α) expression was assessed by *in situ* hybridization and immunofluorescence, respectively. The relationship between fibroblast growth factor pathway marker levels and progression-free survival (PFS) was analyzed using Kaplan-Meier and Cox proportional hazards regression methods.

**Results:**

Univariate analysis indicated that more intense FGFR1 staining was associated with shorter PFS (log-rank *P* = 0.0452), but FRS2α staining was not significantly associated with PFS (log-rank *P* = 0.2610). Multivariate Cox proportional hazards regression models were constructed for FGFR1 and FRS2α individually, adjusting for baseline Eastern Cooperative Oncology Group performance status, treatment arm and anemia status. When adjusted for each of these variables, the highest intensity level of FGFR1 (level 3 or 4) had increased progression risk relative to the lowest intensity level of FGFR1 (level 1) (*P* = 0.0115). The highest intensity level of FRS2α (level 3 or 4) had increased progression risk relative to the lowest intensity level of FRS2α (level 1) (*P* = 0.0126).

**Conclusions:**

Increased expression of FGFR1 and FRS2α was associated with decreased PFS among patients with metastatic RCC treated with sorafenib. The results suggest that FGF pathway activation may impact intrinsic resistance to VEGF receptor inhibition.

**Electronic supplementary material:**

The online version of this article (doi:10.1186/s12885-015-1302-1) contains supplementary material, which is available to authorized users.

## Background

Cancers of the kidney and renal pelvis affect more than 61,000 patients annually and the most common pathological subtype is clear cell renal cell carcinoma (ccRCC) [1]. Over 13,000 patients die annually from RCC, making it one of the top 10 leading causes of cancer deaths. Contemporary treatments include inhibitors of vascular endothelial growth factor receptor (VEGFR) and mammalian target of rapamycin (mTOR); the choice of inhibitors is currently empirical and resistance to treatment typically occurs [2].

Angiogenesis plays a key role in the growth of many tumors and is mediated by growth factors in both the fibroblast growth factor (FGF) and VEGF families [3,4]. Durable clinical responses with current VEGF tyrosine kinase inhibitors (TKIs) may be limited by acquired or intrinsic tumor resistance. Acquired resistance to VEGF blockade in various mouse models appears to be via activation of VEGF-independent pathways with secondary activation of proangiogenic ligands from the FGF family [5,6]. Fibroblast growth factor-2 expression is increased in post-VEGF TKI treatment biopsies further supporting the role of FGF signaling in acquired resistance [7].

There are four FGF tyrosine kinase membrane receptors (FGFRs): FGFR1, −2, −3 and −4 [8]. FGFs bind their receptors to mediate cell proliferation, angiogenesis, and aberrant pathway activation associated with tumor neovascularization. Downstream targets of FGFR signaling include fibroblast growth factor receptor substrate 2 alpha (FRS2α), which promotes cell proliferation [9]. Intrinsic resistance to TKIs is associated with poor clinical outcomes and overexpression of FGFR1,-2 has been observed in RCC [10,11]. However, the impact of expression of FGF signaling components on response to first-line treatment TKIs is unknown. We hypothesized that FGF signaling may impact response to sorafenib, a TKI whose targets include VEGFR2 and platelet-derived growth factor receptor (PDGFR) [12].

Our retrospective analysis of a phase II clinical trial suggest that increased expression of FGFR1 and FRS2α is associated with decreased progression-free survival (PFS) in patients with RCC treated with first-line sorafenib. The results suggest that FGF pathway activation may be associated with intrinsic tumor resistance to sorafenib.

## Methods

### Clinical samples

In a prospective phase II trial, untreated patients with metastatic clear cell RCC were randomly allocated to receive sorafenib 400 mg orally twice daily with or without subcutaneous interferon α (0.5 million units twice daily). Eighty participants were enrolled from June 25, 2005 through June 18, 2007 [13]. Primary endpoints included the objective response rate (ORR) and safety. Secondary endpoints included progression-free survival (PFS) and overall survival (OS). All patients had signed an informed consent approved by The University of Texas MD Anderson Cancer Center institutional review board under protocols 2003–0982 and 2004–0526 (ClinicalTrials.gov Identifier NCT00126594). An experienced genitourinary pathologist (P.T.) centrally reviewed hematoxylin-eosin slides for available patient tumors (n = 40) in order to confirm histological classification and standardize pathologic features.

### Statistical methods

Associations between marker levels and clinical variables (sex, ethnicity, performance status, prognostic risk, and anemia defined as hemoglobin level <14 g/dL for men or <12 g/dL for women) were analyzed using the Fisher’s exact test. PFS was analyzed with regard to marker levels for FGFR1 and FRS2α in a cohort of 40 patients with available nephrectomy specimens. PFS was defined as the number of months from the start of chemotherapy, until a patient’s death or disease progression. Patients whose disease had not progressed were censored at the last follow-up date. Patients whose disease had not progressed before starting a new treatment were censored at the new treatment start date. The relationship between tumor marker levels and PFS was analyzed using Kaplan-Meier and Cox proportional hazards regression methods. Because the phase II study used the Pocock-Simon minimization method [14] to randomize patients to treatment arms, the balancing variables were included in the multivariate models along with the treatment arm [15]. These variables included ECOG performance status (1 vs 0) and baseline anemia (No vs Yes, where Yes is based on Hb < 14 for males and Hb < 12 for females). Although randomization procedures also balanced treatment arms on nephrectomy (No vs Yes) and LDH (Not elevated vs Elevated, where Elevated is > 1.5× ULN), none of the 40 patients in the biomarker study had elevated LDH and all had a nephrectomy; therefore, the multivariate models were not adjusted for these two factors. Other prognostic factors, such as corrected calcium, were not available. All *P-values* were two tailed and considered significant at α < 0.05. Analyses were conducted using SAS for Windows (release 9.1, SAS Institute, Cary, NC, USA).

### Tissue microarrays

Tissue microarrays were generated using an arrayer (Beecher Instruments, Inc., Sun Prairie, WI) with 0.6-mm cores in triplicate for each case. The slides were scanned with the Leica Microsystem (Leica Microsystems Inc. Buffalo Grove, IL) at 20X using the Ariol Scan Station. The Ariol system (Applied Imaging, San Jose, CA) was used to analyze images. Areas of viable tumor were gated by a genitourinary pathologist (P.T.) for analysis; areas of non-viable tumor and non-tumor tissue were excluded. A cytoplasmic algorithm was applied using the multi-stain version of the software. Digitally, the DAB stained cells would be positive and the negative cells stained with hematoxlin would be measured for area. We used TMA Navigator software (Applied Imaging, San Jose, CA) to quantitate the tumor (scale of 0–100) intensity and stratification of biomarkers into quartiles for each core at 20X magnification.

### Histology, *In Situ* Hybridization (ISH), and Immunofluorescence (IF)

Four-micron paraffin sections are cut and dried at room temperature for 30 minutes prior to being placed in the oven at 56°C overnight. The *in situ* hybridization for FGFR1 was performed using a protocol as previously described [16]. Immunofluorescence using primary antibodies (FRS2α) was performed as previously described [17]. TO-PRO-3 was used as a nuclear counterstain. The Ariol imaging platform was used to stratify the specimens based on intensity of staining for FGF biomarkers (FGFR1 ISH, FRS2α IF); the stratification was independently confirmed by a pathologist (P.T.).

## Results

The baseline patient characteristics are denoted in supplemental information, Table 1 for the previously reported phase II clinical trial of first-line sorafenib therapy in metastatic RCC [13]. Seventy-three percent of study participants were male. Participants had an ECOG status of 0 (68%) or 1 (32%), and all had a Memorial Sloan-Kettering Cancer Center (MSKCC) prognostic risk of low or intermediate except for 1 patient categorized as poor and 1 patient with missing data. Race/ethnicity was white, non-Hispanic for 80% and Hispanic, Black, or Native American for 20% (Additional file 1: Table S1). At baseline, 37% of the patients had anemia and 63% did not. Patient age at registration ranged from 45 to 83 years of age (mean 62.38, SD 8.59).

**Table 1:** **Univariate cox proportional hazards regression models of progression free survival from chemotherapy start**

Variable	Progressed	Total	HR	95% CI	P
**PFS (29 of 40 progressed)**					
**Treatment arm**					
**Sorafenib**	19	22	2.05	0.95, 4.45	0.0683
**Sorafenib + interferon**	10	18	1.00		
**ECOG performance status**					
**1**	10	13	4.95	2.07, 11.84	0.0003
**0**	19	27	1.00		
**Anemia**					
**Yes**	12	15	2.37	1.09, 5.17	0.0299
**No**	17	25	1.00		
**FGFR1**					
**(level 1)**	3	8	1.00		
**(level 2)**	15	19	3.54	1.02, 12.29	0.0463
**(level 3 or 4)**	11	13	4.56	1.25, 16.66	0.0218
**FRS2**α					
**(level 1)**	3	7	1.00		
**(level 2)**	17	21	2.07	0.60, 7.18	0.2498
**(level 3 or 4)**	9	12	2.87	0.77, 10.66	0.1146

ECOG, Eastern Cooperative Oncology Group; FGFR1, fibroblast growth factor receptor 1; FRS2α, fibroblast growth factor receptor substrate 2 alpha; HR, hazard ratio.

**Figure 1:**
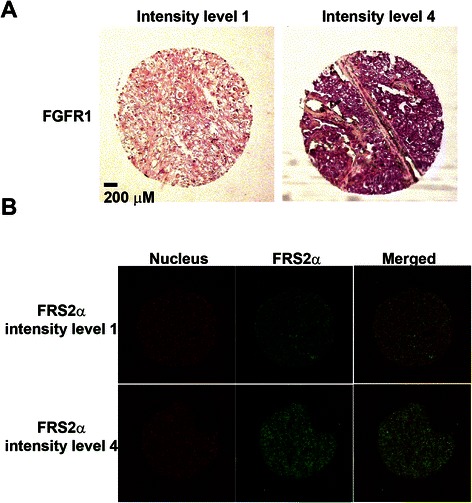
Fibroblast growth factor receptor 1 and fibroblast growth factor receptor substrate 2 alpha expression. Intensity levels were stratified using the Ariol imaging platform (higher number corresponds to higher intensity) in renal cell carcinoma. Non-tumor tissue was excluded from analysis. **(A)***In situ* hybridization for localization of FGFR1 message. **(B)** FRS2α immunofluorescence.

To examine the relationship between expression of FGF biomarkers and PFS, a tissue microarray representing clear cell RCC specimens from patients with available tumor and enrolled on the trial was constructed. Expression of FGFR1 and FRS2α were analyzed by ISH/IF and stratified into 3 categories (low, level 1; intermediate, level 2; high, level 3 or 4) (Figure 1). We did not observe an association with FGFR1 intensity with patient characteristics (sex, ethnicity, ECOG performance status, MSKCC prognostic risk category or anemia). Only baseline anemia was associated with FRS2α intensity (*P* = 0.0168), Additional file 2: Table S2 and Additional file 3: Table S3.

**Figure 2:**
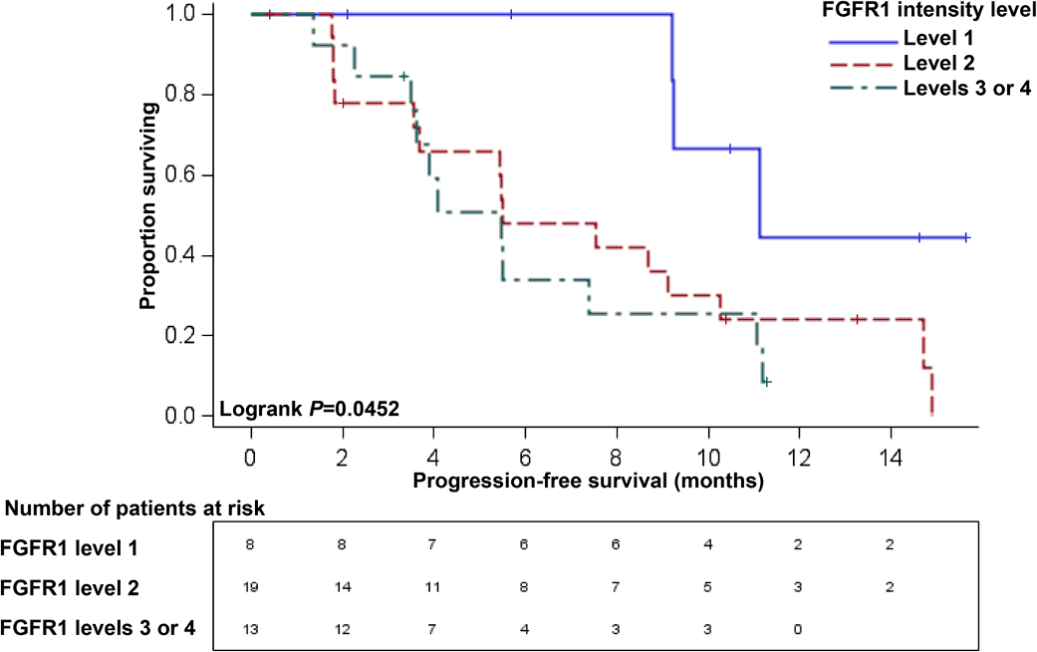
Progression-free survival curves (PFS) with number at risk stratified by FGFR1 intensity. The Ariol imaging platform was used to stratify the specimens based on intensity of *in situ* hybridization staining for FGFR1. Non-tumor tissue was excluded from analysis. The differences across FGFR1 intensity strata were statistically significant with better progression-free survival among those patients with the lowest FGFR1 intensity (level 1) in univariate analysis; *P* = 0.0452.

**Figure 3:**
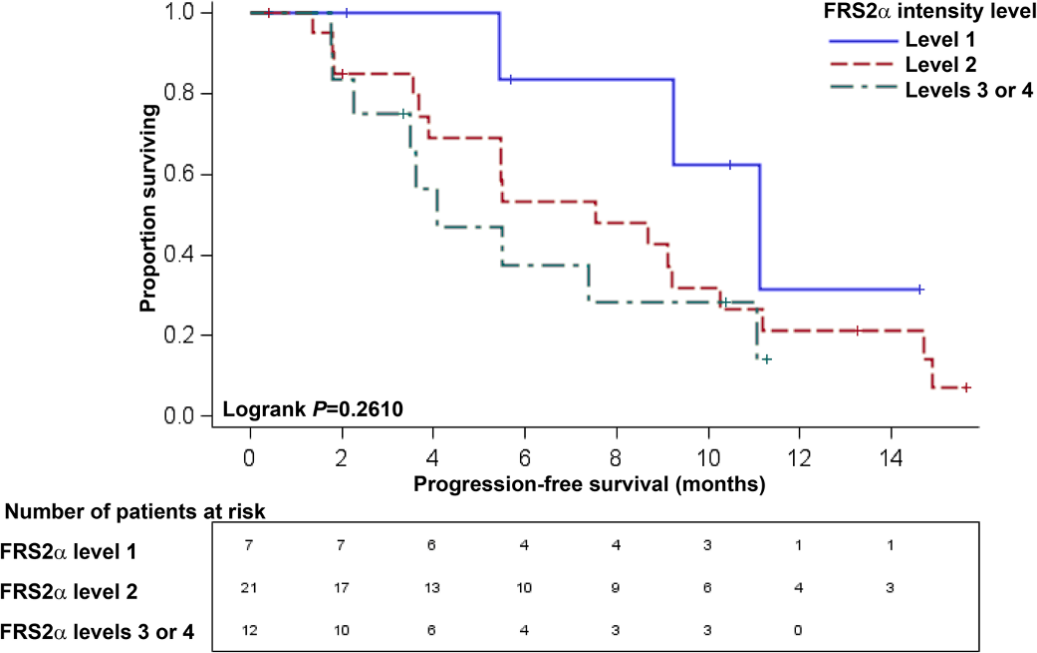
Progression-free survival curves (PFS) with number at risk stratified by FRS2α intensity. The Ariol imaging platform was used to stratify the specimens based on intensity of immunofluorescence staining for FRS2α. Non-tumor tissue was excluded from analysis. The differences across FRS2α intensity strata were not statistically significant in univariate analysis; *P* = 0.2610.

In univariate models, PFS was associated with ECOG performance status (*P* = 0.0003), anemia (*P* = 0.0299) and FGFR1 expression (*P* = 0.0218), Table 1. The median PFS for the total sample was 7.56 (95% CI 4.07 to 10.25) months. Median PFS by FGFR1 intensity was 5.49 (95% CI 3.48 to 11.07) months for those with the highest intensities (level 3 or 4), 5.52 (95% CI 3.55 to 10.25) months for those with intermediate intensity (level 2), and 11.14 (95% CI 9.20, not attained) months for those with the lowest FGFR1 intensity (level 1). Similarly, median PFS by FRS2α intensity was 4.07 (95% CI 1.81 to 11.07) months for those with the highest intensities (level 3 or 4), 7.56 (95% CI 3.68 to 10.25) months for those with intermediate intensity (level 2), and 11.14 (95% CI 5.45, not attained) months for those with lowest FRS2α intensity (level 1). Kaplan-Meier curves indicated that more intense FGFR1 staining was associated with shorter PFS (*P* = 0.0452), but FRS2α staining was not significantly associated with PFS (*P* = 0.2610) (Figures 2 and 3).

ECOG performance status, baseline anemia, nephrectomy and LDH were balancing variables used during the randomization of patients to treatment arm. Multivariate Cox proportional hazards regression models were constructed for FGFR1 and FRS2α individually, adjusting for baseline ECOG performance status, treatment arm and baseline anemia (Tables 2 and 3). When adjusted for each of these factors, the risk of progression was significantly higher for tumors with the highest intensity level of FGFR1 (level 3 or 4) (HR 5.92, 95% CI 1.49 to 23.56, *P* = 0.0115) and for those with an intermediate intensity level of FGFR1 (level 2) (HR 4.21, 95% CI 1.13 to 15.72, *P* = 0.0326) relative to the risk among those with the lowest intensity level of FGFR1 (level 1). Tumors with the highest intensity level of FRS2α (level 3 or 4) had increased risk of progression relative to those with the lowest intensity level of FRS2α (level 1) (HR 7.32, 95% CI 1.53 to 34.97, *P* = 0.0126); progression risk did not differ significantly between those with intermediate versus low FRS2α intensity (level 2 vs. level 1) (HR 2.96, 95% CI 0.76 to 11.45, *P* = 0.1161). Our data indicates that increased expression of FGFR1 and FRS2α is associated with a worse PFS on first-line sorafenib treatment.

**Table 2:** **Multivariate cox proportional hazards regression model to predict progression-free survival from chemotherapy start by FGFR1 intensity level**

Variable	Level	Hazard ratio	95% Hazard ratio confidence limits		Probability > Chi square test
**Treatment arm**	Sorafenib vs. Sorafenib + interferon	1.581	0.697	3.585	0.2725
**ECOG Status**	1 vs. 0	4.630	1.417	15.134	0.0112
**Anemia**	Yes vs. No	1.481	0.531	4.131	0.4535
**FGFR1 Intensity**	(3 or 4) vs. 1	5.925	1.490	23.561	0.0115
	2 vs. 1	4.209	1.126	15.725	0.0326

ECOG, Eastern Cooperative Oncology Group; FGFR1, fibroblast growth factor receptor 1.

**Table 3:** **Multivariate cox proportional hazards regression model to predict progression-free survival from chemotherapy start by FRS2α intensity level**

Variable	Level	Hazard ratio	95% Hazard ratio confidence limits		Probability > Chi square test
**Treatment arm**	Sorafenib vs. Sorafenib + interferon	1.915	0.850	4.314	0.1171
**ECOG Status**	1 vs. 0	4.651	1.508	14.341	0.0075
**Anemia**	Yes vs. No	1.831	0.628	5.342	0.2683
**FRS2α Intensity**	(3 or 4) vs. 1	7.318	1.531	34.971	0.0126
	2 vs. 1	2.959	0.765	11.449	0.1161

ECOG, Eastern Cooperative Oncology Group; FRS2α, fibroblast growth factor receptor substrate 2 alpha.

## Discussion

Patients that do not initially benefit from tyrosine kinase inhibition may have intrinsically resistant tumors. We investigated expression of FGF pathway components in patients with metastatic RCC treated in a randomized phase II clinical trial that yielded no differences in PFS between sorafenib versus sorafenib + interferon α [13]. In our current study, expression of FGFR1 and FRS2α was not associated with MKSCC prognostic risk categories. Using univariate and multivariate analyses, we observed that increased expression of FGFR1 is associated with a shorter PFS in patients treated with first-line sorafenib. In the phase II trial design, to balance the prognostic factors between treatment arms, the Pocock-Simon minimization method was used to randomize patients and balance prognostic factors such as ECOG performance status, baseline anemia, nephrectomy, and LDH. The highest FRS2α expression levels were associated with a shorter PFS after adjustment for study treatment, baseline anemia or performance status.

Activation of VEGF-independent pathways such as FGF signaling can promote cell proliferation, cell migration and tumorigenesis [5,6]. VEGFR2 blockade transiently stops tumor growth and is followed by tumor progression and restoration of tumor-associated endothelium. Blockade of the FGF pathway using adenoviral delivery of a soluble FGFR2, which acts as a FGF-trap, decreases secondary angiogenesis after VEGFR2 blockade suggesting that FGF inhibition may block acquired VEGF-independent pathways [5]. Consistent with this hypothesis, soluble high affinity decoy FGF receptors inhibit cell proliferation and treatment with a FGF ligand trap blocks VEGF-independent reactivation of tumor angiogenesis in a mouse model of pancreatic neuroendocrine tumors [4,18].

All currently approved targeted therapies for metastatic RCC (sunitinib, sorafenib, temsirolimus, everolimus, bevacizumab, pazopanib and axitinib) lack FGFR activity [19]. In a phase II study of dovitinib, a TKI that inhibits FGFR and VEGFR, as second-line therapy in metastatic RCC, the disease control rate ≥ 4 months was 51% [20]. However, no amplification of FGFR1,-2,-3 by PCR was detected in archival tissue suggesting that PCR may not accurately reflect FGF activation after progression on first-line therapy. In a phase III trial comparing dovitinib with sorafenib as third-line targeted therapy in metastatic RCC, there were no differences in efficacy outcomes suggesting that there may be other mechanisms of VEGF-targeted therapy than FGF activation [21].

Our study has several limitations. Although the samples were part of a randomized clinical trial, our sample size (N = 40) was small in comparison to prior studies [20,21] as tissue was not available from all enrolled patients, limiting the validity of the observations to the cohort. We did not directly compare our ISH/IF assay with other FGF assays such as PCR amplification of FGFR or FGF serum levels. RCC has intratumoral heterogeneity and we attempted to control for macroscopic heterogeneity by using the Ariol platform to exclude stroma and stratify the ISH/IF intensities, however it is unknown whether FGFR1/FRS2α expression in FFPE nephrectomy samples is concordant with expression in distant metastases [22,23]. The Ariol platform, as an automated quantitative analysis, has been studied with other biomarkers with concordance between manual and automated scoring [24], however it is unknown the limitations of our IHC/IF assay with respect to formalin-fixed paraffin-embedded age or fixation conditions. The study was retrospective and FGF activation was not used in the initial stratification of treatment arms. There are also likely additional mechanisms beyond FGF activation [25] that contribute to intrinsic TKI resistance and we did not evaluate other commonly used TKIs such as pazopanib or sunitinib. Future investigation is warranted to determine whether these markers are associated with response to other TKIs.

The results from our study, which used FGFR1 ISH and FRS2α IF to assess FGF activation pre-treatment, could be used to build predictive models in RCC to prospectively identify patients who would benefit from therapeutic strategies targeting both VEGF and FGF signaling in the first-line setting. FGF pathway activation may contribute to intrinsic VEGF TKI resistance, or angiogenesis in tumors with FGF activation may be less dependent on VEGF-mediated pathways. Predictive biomarkers of intrinsic VEGF TKI resistance are lacking and the efficacy of dual inhibition of FGF and VEGF in the subset of patients with increased FGFR1 expression prior to treatment requires further study.

## Conclusions

Increased expression of FGFR1 or FRSα was associated with decreased PFS in a phase II clinical trial of patients with metastatic RCC treated with first-line sorafenib. The results suggest that FGF pathway activation may be associated with an intrinsic resistance to VEGF receptor inhibition.

## Additional files

Additional file 1: Table S1. Patient characteristics.
Additional file 2: Table S2. Association of FGFR1 Intensity With Patient Characteristics.
Additional file 3: Table S3. Association of FRS2α Intensity With Patient Characteristics.

## Abbreviations

ccRCC: Clear cell renal cell carcinoma
ECOG: Eastern Cooperative Oncology Group
FGF: Fibroblast growth factor
FGFR1: Fibroblast growth factor receptor 1
FRS2α: Fibroblast growth factor receptor substrate 2 alpha
IF: Immunofluorescence
ISH: In situ hybridization
MKSCC: Memorial Sloan-Kettering Cancer Center
ORR: Objective response rate
PDGFR: Platelet-derived growth factor
PFS: Progression-free survival
TKI: Tyrosine kinase inhibitor
VEGF: Vascular endothelial growth factor
