# Supplementary material for: The impact of FGFR1 and FRS2α expression on sorafenib treatment in metastatic renal cell carcinoma
Source: BMC Cancer. 2015 Apr 18;15:304. doi: 10.1186/s12885-015-1302-1 (PMC4406182; doi:10.1186/s12885-015-1302-1)
Supplement: Additional file 1: Table S1. — Patient characteristics. [file 12885_2015_1302_MOESM1_ESM.docx]

**Additional file 1: Table S1.** Patient characteristics.

| **Characteristic** | **Number of patients (*n*=40)** | **(%)** |
| --- | --- | --- |
| **Sex** |  |  |
| **Male** | 29 | (73) |
| **Female** | 11 | (27) |
| **Ethnicity** |  |  |
| **White, Non-Hispanic** | 32 | (80) |
| **Hispanic, Black, or**  **Native American** | 8 | (20) |
| **ECOG status** |  |  |
| **0** | 27 | (68) |
| **1** | 13 | (32) |
| **MSKCC prognostic risk** |  |  |
| **Low** | 21 | (54) |
| **Intermediate** | 17 | (44) |
| **Poor** | 1 | (2) |
| **Missing** | 1 |  |
| **Treatment Arm** |  |  |
| **Sorafenib** | 22 | (55) |
| **Sorafenib + interferon** | 18 | (45) |

ECOG, Eastern Cooperative Oncology Group; MSKCC, Memorial Sloan-Kettering Cancer Center.
