# Supplementary material for: The impact of FGFR1 and FRS2α expression on sorafenib treatment in metastatic renal cell carcinoma
Source: BMC Cancer. 2015 Apr 18;15:304. doi: 10.1186/s12885-015-1302-1 (PMC4406182; doi:10.1186/s12885-015-1302-1)
Supplement: Additional file 2: Table S2. — Association of FGFR1 Intensity With Patient Characteristics. [file 12885_2015_1302_MOESM2_ESM.docx]

**Additional file 2: Table S2.** Association of FGFR1 Intensity With Patient Characteristics

| Variable | Level | FGFR1 Intensity | | | | | | *P |
| --- | --- | --- | --- | --- | --- | --- | --- | --- |
|  |  | 1 | | 2 | | 3 or 4 | |  |
|  |  | n | (%) | n | (%) | n | (%) |  |
| Sex | Male | 4 | (50.00) | 14 | (73.68) | 11 | (84.62) | 0.2658 |
|  | Female | 4 | (50.00) | 5 | (26.32) | 2 | (15.38) |  |
| Race/ethnicity | White, Non-Hispanic | 6 | (75.00) | 16 | (84.21) | 10 | (76.92) | 0.7746 |
|  | Hispanic, Black, or Native American | 2 | (25.00) | 3 | (15.79) | 3 | (23.08) |  |
| ECOG Performance Status | 0 | 7 | (87.50) | 12 | (63.16) | 8 | (61.54) | 0.4177 |
|  | 1 | 1 | (12.50) | 7 | (36.84) | 5 | (38.46) |  |
| Baseline anemia | No | 5 | (62.50) | 10 | (52.63) | 10 | (76.92) | 0.4347 |
|  | Yes | 3 | (37.50) | 9 | (47.37) | 3 | (23.08) |  |
| MSKCC prognostic risk | Good | 4 | (50.00) | 9 | (50.00) | 8 | (61.54) | 0.6824 |
|  | Intermediate | 4 | (50.00) | 9 | (50.00) | 4 | (30.77) |  |
|  | Poor | 0 | (0.00) | 0 | (0.00) | 1 | (7.69) |  |
|  | *Missing (n = 1)* |  |  |  |  |  |  |  |
| Age at study enrollment (yrs) | mean (SD) | 62.38 | (8.28) | 63.11 | (7.89) | 61.31 | 10.21 | ^α^ 0.7253 |
| Age at diagnosis (yrs) | mean (SD) | 60.63 | (7.91) | 61.11 | (7.26) | 57.54 | 12.20 | ^α^ 0.3881 |

**P*-values are from Fisher’s exact test unless otherwise noted.

α *P*-value from one-way ANOVA, between groups design.

ECOG, Eastern Cooperative Oncology Group; FGFR1, fibroblast growth factor receptor 1.
