# Supplementary material for: The impact of FGFR1 and FRS2α expression on sorafenib treatment in metastatic renal cell carcinoma
Source: BMC Cancer. 2015 Apr 18;15:304. doi: 10.1186/s12885-015-1302-1 (PMC4406182; doi:10.1186/s12885-015-1302-1)
Supplement: Additional file 3: Table S3. — Association of FRS2α Intensity With Patient Characteristics. [file 12885_2015_1302_MOESM3_ESM.docx]

**Additional file 3: Table S3.** Association of FRS2α Intensity With Patient Characteristics

| Variable | Level | FRS2α Intensity | | | | | | *P |
| --- | --- | --- | --- | --- | --- | --- | --- | --- |
|  |  | 1 | | 2 | | 3 or 4 | |  |
|  |  | n | (%) | n | (%) | n | (%) |  |
| Sex | Male | 4 | (57.14) | 18 | (85.71) | 7 | (58.33) | 0.1090 |
|  | Female | 3 | (42.86) | 3 | (14.29) | 5 | (41.67) |  |
| Race/ethnicity | White, Non-Hispanic | 5 | (71.43) | 17 | (80.95) | 10 | (83.33) | 0.7579 |
|  | Hispanic, Black, or Native American | 2 | (28.57) | 4 | (19.05) | 2 | (16.67) |  |
| ECOG Performance Status | 0 | 6 | (85.71) | 13 | (61.90) | 8 | (66.67) | 0.5939 |
|  | 1 | 1 | (14.29) | 8 | (38.10) | 4 | (33.33) |  |
| Baseline anemia | No | 5 | (71.43) | 9 | (42.86) | 11 | (91.67) | 0.0168 |
|  | Yes | 2 | (28.57) | 12 | (57.14) | 1 | (8.33) |  |
| MSKCC prognostic risk | Good | 4 | (57.14) | 10 | (50.00) | 7 | (58.33) | 1.0000 |
|  | Intermediate | 3 | (42.86) | 9 | (45.00) | 5 | (41.67) |  |
|  | Poor | 0 | (0.00) | 1 | (5.00) | 0 | (0.00) |  |
|  | *Missing (n = 1)* |  |  |  |  |  |  |  |
| Age at study enrollment (yrs) | mean (SD) | 61.57 | (7.79) | 63.10 | (8.80) | 61.58 | (9.24) | ^α^ 0.9178 |
| Age at diagnosis (yrs) | mean (SD) | 60.00 | (7.57) | 61.24 | (8.70) | 57.33 | (10.89) | ^α^ 0.4337 |

**P*-values are from Fisher’s exact test unless otherwise noted.

α *P*-value from one-way ANOVA, between groups design

ECOG, Eastern Cooperative Oncology Group; FRS2α, fibroblast growth factor receptor substrate 2 alpha.
